# Occlusal Caries Detection with Intraoral Scanners in Pediatric Dentistry: A Comparative Clinical Study

**DOI:** 10.3390/jcm13040925

**Published:** 2024-02-06

**Authors:** Nelly Schulz-Weidner, Marina Gruber, Bernd Wöstmann, Constanze Friederike Uebereck, Norbert Krämer, Maximiliane Amelie Schlenz

**Affiliations:** 1 Department of Pediatric Dentistry, Dental Clinic, Justus Liebig University, Schlangenzahl 14, 35392 Giessen, Germany; nelly.schulz-weidner@dentist.med.uni-giessen.de (N.S.-W.);; 2 Department of Prosthodontics, Dental Clinic, Justus Liebig University, Schlangenzahl 14, 35392 Giessen, Germanybernd.woestmann@dentist.med.uni-giessen.de (B.W.)

**Keywords:** intraoral scanners, dental caries, occlusal caries detection, clinical study, digital dentistry, pediatric dentistry, interdisciplinary study, occlusal caries, primary teeth, permanent teeth

## Abstract

**Background:** The aim of this clinical study was to compare the occlusal caries detection (OCD) performance of the intraoral scanners (IOSs) Trios 4 (TIO, 3Shape) and Emerald S (EME, Planmeca) and the Diagnocam (DIA, KaVo) with the established visual (WHO) examination (VIS, reference method). **Methods:** Between 08/2022 and 02/2023, 60 children (mean age 9.6 ± 2.5 years) were examined as part of their regular dental checkups. OCD was performed at the tooth level, separately for primary and permanent unrestored teeth. Furthermore, two thresholds were analyzed: sound versus overall caries (pooled data of enamel and dentin caries, TH1) and pooled data of sound and enamel caries versus dentin caries (TH2). **Results:** The best agreement with the reference method (reliability) in both dentitions was obtained for DIA (ĸ = 0.829/ĸ = 0.846; primary/permanent teeth), followed by EME (ĸ = 0.827/ĸ = 0.837) and TIO (ĸ = 0.714/ĸ = 0.680). Similar results were shown for the diagnostic quality (sensitivity, specificity and area under the curve of the receiver operating characteristic curve), with higher values for TH1 than for TH2. Both IOSs and the DIA showed worse results than the reference method VIS. **Conclusions:** Currently, IOS should be used as an additional caries detection tool, especially for visualization, and cannot be recommended as a basic tool for diagnosis or invasive/noninvasive therapy decisions in OCD.

## 1. Introduction

The use of intraoral scanners (IOSs) beyond impression taking is a growing field in dentistry. Colored intraoral scans might be used for shade selection [1] or plaque monitoring [2], and monitoring software allows detection of tooth movement or wear at the micrometer level [3]. In addition to these applications, some IOSs offer a caries detection tool based on near-infrared light transillumination or fluorescence technology that could enable the possible detection of proximal and/or occlusal carious lesions in unrestored teeth [1,4,5]. Therefore, in pediatric dentistry in particular, this method might be very promising as an additional caries detection tool in routine dental checkups or dental screening in kindergartens or schools, especially regarding the visualization of carious lesions. The advantage of an IOS is that the findings can be displayed visually on a screen and easily stored for later comparison with older intraoral scans. Therefore, it could be hypothesized that carious lesions would be easier to visualize and document in daily patient care.

Visual examination is the primary caries detection method in daily dental practice. Nevertheless, it also underestimates the caries burden on occlusal surfaces [6,7], and there is a consensus that the dentist needs to use additional caries detection methods [8]. It is important to distinguish between enamel and dentinal caries lesions in order to differentiate between preventive and invasive treatment options [9]. For this reason, more studies are evaluating innovative methods of caries detection and quantification to help dentists to detect caries and demineralization at an early stage [10,11]. Regarding occlusal caries, visual examination is the preferred method [12]. Nevertheless studies have shown that near-infrared transillumination, as used in the Diagnocam (KaVo, Bieberach, Germany), can be effective in detecting hidden occlusal caries in primary and permanent teeth without previous treatment such as fillings or other restorations [13,14,15]. By using a wavelength of 780 nm, light scattering is reduced, allowing deeper penetration into the tissue. As a result, in both dentitions, sufficient contrast between a carious lesion and the surrounding sound hard tissue is recorded. Increased pore volume in demineralized enamel leads to changes in its optical properties. Therefore, more fluorophores are detected showing fluorescence of the lesion, the porphyrins produced by microbial metabolism [16,17]. In addition, the fluorescence method has been recommended for use only as an adjunct to visual examination, as it has been reported to yield more false-positive results than visual and radiographic methods [18]. Although it appears to be able to detect caries lesions at an earlier stage, it seems to have high rates of misclassifying sound tooth surfaces as carious [19]. In combination with visual inspection, the method is mainly used as a valuable adjunct to a second opinion [20]. Caries detection in primary teeth with additional tools might be made easier due to tooth morphology of thin enamel and dentine where the tooth morphology already presents the challenges of thin enamel and dentine and relatively large pulp chambers [21].

Little is known about the clinical performance of intraoral-scanner-based caries detection, particularly with regard to differentiating between enamel and dentinal caries lesions. A limitation of the IOS as a light-optical system is that it cannot detect subgingival carious lesions and teeth with restorations and sealants with secondary caries. Moreover, detecting of cracks is not possible. A previous in vitro study on extracted unrestored primary and permanent teeth systematically investigated the three commercially available IOSs with caries detection tools—iTero Element 5D (Align Technology, San José, CA, USA) and Emerald S (Planmeca, Helsinki, Finland), which use near-infrared light transillumination for caries detection, and Trios 4 (3Shape, Copenhagen, Denmark), which uses fluorescence technology for caries detection—as well as the established methods—Diagnocam, which uses near-infrared light transillumination for caries detection, bitewing radiography and visual examination. The results were compared with microcomputed tomography (µ-CT) images of the extracted teeth. Although no single method could be generally recommended for the detection of proximal and occlusal carious lesions in both dentitions, IOSs showed in vitro in some cases even better results than established caries detection methods [5]. However, to the best of the authors’ knowledge, only one clinical study of proximal caries detection comparing bitewing radiography and iTero Element 5D has been described [22]. The difficulty with clinical caries assessment is that µ-CT images or histological sections of teeth cannot be obtained without tooth extraction. Therefore, only bitewing radiography or visual examination of teeth can be used as a reference method, depending on whether proximal or occlusal caries detection is being assessed.

The aim of this clinical study was to evaluate the new intraoral-scanner-based caries detection tools of the IOSs Trios 4 and Emerald S and also using the established Diagnocam, with respect to occlusal caries detection in posterior teeth without sealants or restorations in both dentitions. The results were compared with the gold standard visual examination as a reference. The null hypothesis was formulated that there would be no significant difference between visual examination and Diagnocam, Trios 4 or Emerald S in terms of reliability, sensitivity and specificity for the detection of enamel and dentinal caries lesions.

## 2. Materials and Methods

### 2.1. Study Design

This comparative clinical study evaluated four different methods of occlusal caries detection in children as part of the regular dental checkup. First, the established visual examination was performed as a reference method for later analysis. Then, a Diagnocam (version 2.4.2.6944, KaVo, Biberach, Germany) and the new intraoral-scanner-based methods Trios 4 (version 20.1.4, 3Shape, Copenhagen, Denmark) and Emerald S (version 6.0.1.812, Planmeca, Helsinki, Finland) were used.

**Figure 1:**
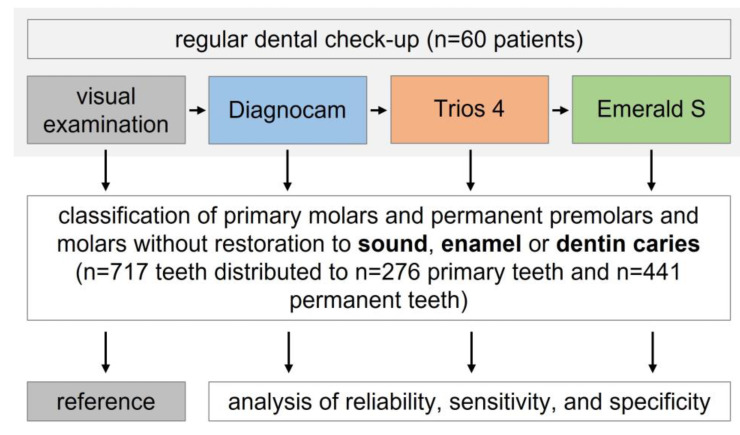
Flow scheme of the clinical study.

The evaluation of occlusal caries detection was performed at the tooth level. Only primary molars and permanent premolars and molars of the upper and lower jaw were included, while incisors and canines or teeth treated with silver diamine fluoride, sealants, fillings or crowns were excluded. The results are presented separately for the primary and the permanent teeth. A flowchart of the clinical study is shown in Figure 1.

### 2.2. Study Group

All children between the ages of 5 and 14 (mean age 9.6 ± 2.5 years) who attended a regular dental checkup at the Department of Pediatric Dentistry of the Justus Liebig University Giessen (JLU, Germany) between August 2022 and February 2023 were invited to participate in this clinical study. However, children with serious general illnesses and those whose parents objected to dental screening were not included. In addition to a detailed verbal explanation of the study procedure, written informed consent was obtained from all parents/primary caregivers (PGs). The children and their accompanying PGs were informed about the dental findings, and if necessary, all appropriate therapeutic options were enumerated and offered.

This study was conducted according to the guidelines of the Declaration of Helsinki, approved by the Ethics Committee of the Medical Faculty of the JLU (ref. no. 46/20) and registered in the German Clinical Trial Register (DRKS00029333).

### 2.3. Standardization, Calibration and Blinding

To ensure comparable test conditions and to obtain reproducible data according to predefined criteria, two experienced operators (N.S.-W. and C.F.U.) performed the visual examination for the occlusal caries detection, which was defined as the reference method for later analysis [23]. Subsequently, a third operator (M.G.) performed the examination with the Diagnocam, Trios 4 and Emerald S.

Prior to this study, the three operators conducting the examination were thoroughly trained by experts in each field. The training included a theoretical part in which the criteria for caries detection were explained. A practical exercise was carried out on a test model with extracted primary and permanent teeth with sound and enamel and dentinal caries lesions. A specialist in pediatric dentistry (N.S.-W.) with 20 years of clinical experience calibrated the two established caries detection methods (visual examination and Diagnocam). For the intraoral scanner-based caries detection, an expert in intraoral scanning (M.A.S.) gave instructions. This allowed the determination of intra-rater reliability (agreement between the findings of each examiner) and inter-rater reliability (agreement between the diagnoses of different examiners) [8]. According to Landis and Koch [24], the level of agreement between operators and trainers was almost perfect (Cohen’s kappa coefficient (κ) > 0.81). The IOSs were calibrated prior to scanning according to the manufacturer’s instructions.

After six weeks, all images from the Diagnocam, Trios 4 and Emerald S were randomly examined for carious lesions by the same operator (M.G.), who waited one week between each method. Statistical analysis and pooling of the results of the caries detection methods was performed by another operator (M.A.S.) who was not involved in the clinical data collection.

### 2.4. Caries Detection Methods

**Figure 2:**
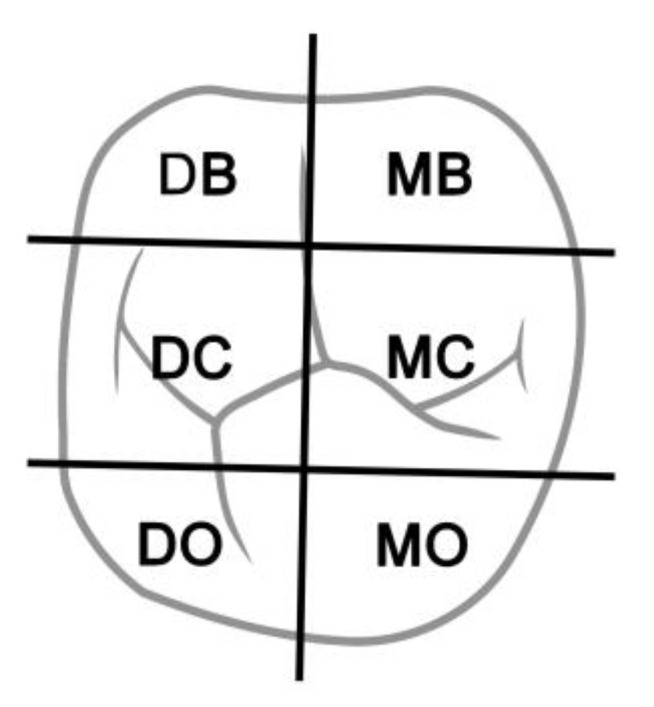
Example of the occlusal surface of a molar divided into the six areas: mesiobuccal (MB), distobuccal (DB), mesiocentral (MC), distocentral (DC), mesiooral (MO) and distooral (DO).

For comparison, standardized measurement was implemented. For this, the occlusal surface of each tooth was divided into six areas: mesiobuccal (MB), distobuccal (DB), mesiocentral (MC), distocentral (DC), mesiooral (MO) and distooral (DO, Figure 2). Evaluation was carried out separately for primary and permanent teeth.

#### 2.4.1. Visual Examination

For visual examination, a standardized illumination (25,000 lx) was ensured and a dental mirror and an air syringe according to World Health Organization (WHO) criteria along with the decayed-, missing-, filled-teeth (DMF-T/dmf-t) index were used. The surfaces were graded according to the modified classification of Schaefer et al. [8]. In addition, for comparison with the other caries detection methods, sound teeth without visually detectable carious lesions were scored as 0, surfaces with enamel caries lesions were scored as 1 and surfaces with dentinal caries lesions were scored as 2.

#### 2.4.2. Diagnocam

In addition to visual examinations, caries detection was performed using a Diagnocam (software version 2.4.2.8000). This device uses near-infrared light transillumination and operates at a wavelength of 780 nm [25]. Similar to the visual examination, sound surfaces without visible carious lesions in the Diagnocam images were scored as 0, whereas enamel caries lesions were scored as 1 and surfaces with dentinal caries lesions were scored as 2.

#### 2.4.3. Trios 4

The IOS Trios 4 was studied as one of the two intraoral-scanner-based caries detection tools. In contrast to the Emerald S, the same scanning tip can be used for 3D scanning and caries detection. Thus, a full-arch scan was performed following a standardized scan path that included the occlusal surface, oral surfaces and buccal surfaces. The Trios 4 uses fluorescence technology for caries detection at a wavelength of 415 nm. The fluorescence excitation of porphyrin derivatives, which are not present in normal healthy tooth substance, may be a useful marker for bacteria associated with dental caries [26,27].

In the caries detection mode, the IOS software (version 20.1.4) displays the carious lesions on a separate IOS scan, highlighting the initial carious lesions in yellow, while moderate to extensive lesions are displayed in red. Therefore, caries detection is not based on the color intraoral scans but on the fluorescence images. To compare the color scale with the other caries detection methods, sound surfaces without any color were scored as 0, yellow areas showing enamel caries lesions were scored as 1 and red areas showing dentinal caries lesions were scored as 2.

#### 2.4.4. Emerald S

The IOS Emerald S (software version 6.0.1.812) requires a separate “cariosity tip” for detection of carious lesions. The caries detection tool is based on the same technology as the Diagnocam, in particular near-infrared light transillumination, but with a shorter wavelength of 727 nm. This near-infrared light penetrates the more transparent enamel and is strongly scattered by enamel lesions as well as dentine. The backscattered light makes these areas appear to be whiter than sound enamel. The reflection of the infrared light is higher, and the lower the translucency, the brighter the structure [28,29].

Images of the near-infrared light transillumination are displayed on the IOS screen as additional information to the three-dimensional data set of the entire arch. Therefore, caries detection is not based on the color intraoral scans but on the near-infrared transillumination images.

The IOS software does not describe or highlight a classification for carious lesions. Therefore, carious lesions without a translucent spot were scored as 0, carious lesions limited to enamel were scored as 1 and dentinal caries lesions were scored as 2.

### 2.5. Statistical Analysis

IBM SPSS Statistics (version 28, IBM, Armonk, NY, USA) was used for the statistical analysis. Due to the study design of an examination at the tooth level, data were further analyzed separately for primary and permanent teeth.

#### 2.5.1. Reliability

For each caries detection method, agreement was calculated using Gwet’s AC1 (ĸ) [30]. Kappa values were categorized as poor (<0.00), slight (0.00–0.20), fair (0.21–0.40), moderate (0.41–0.60), substantial (0.61–0.80) and almost perfect agreement (0.81–1.00) [14]. The values of sound (0), enamel lesion (1) and dentinal lesion (2) were assumed to be nominally scaled categorical variables.

#### 2.5.2. Sensitivity and Specificity

To analyze the diagnostic quality of each method, the sensitivity, specificity and area-under-curve (AUC) values were determined using receiver-operating-characteristic(ROC) curve analysis, conducting multiple comparisons between the ROC from the different methods and thresholds. The threshold values were determined as follows:

- Threshold I: sound (0) versus overall caries (pooled data of enamel (1) and dentin caries (2)).
- Threshold II: pooled data of sound (0) and enamel caries (1) versus dentin caries (2).

The higher the AUC values, the better the diagnostic quality [31].

## 3. Results

A total of 717 teeth were observed, resulting in 4302 areas (717 teeth × 6 areas) to be examined, distributed to 276 primary teeth and 441 permanent teeth.

### 3.1. Reliability

**Figure 3:**
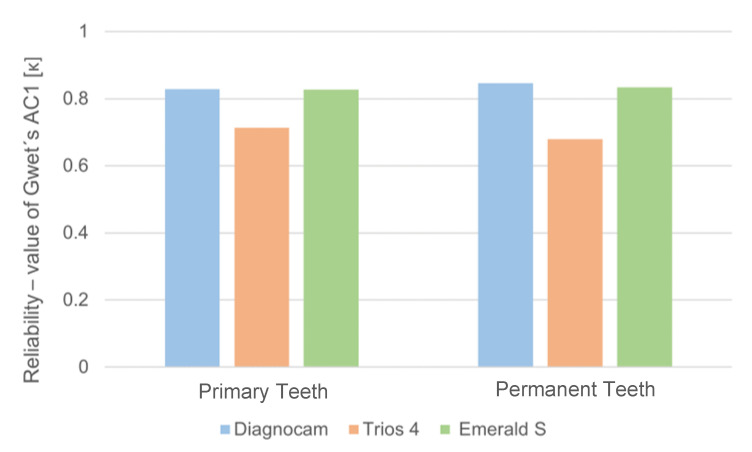
Bar graph of the reliability of the different occlusal caries detection methods distributed to primary and permanent teeth.

For occlusal caries detection in primary teeth, the Diagnocam showed an almost perfect agreement with the reference method visual examination (ĸ = 0.829), followed by the Emerald S (ĸ = 0.827) and the Trios 4 (ĸ = 0.714). These findings were confirmed for the occlusal caries detection in permanent teeth, with the Diagnocam achieving the best agreement with visual examination (ĸ = 0.846). The Emerald S also displayed almost perfect agreement (ĸ = 0.837), followed by the Trios 4 with substantial agreement (ĸ = 0.680, Figure 3).

### 3.2. Sensitivity and Specificity

For threshold I (sound (0) versus overall caries (pooled data of enamel (1) and dentin caries (2))), the sensitivity, specificity and AUC values of near-infrared transillumination (Diagnocam and Emerald S) showed higher values than the fluorescence technology of the Trios 4 in primary and permanent teeth.

For threshold II (pooled data of sound (0) and enamel caries (1) versus dentin caries (2)), the Emerald S showed the highest sensitivity and AUC value and the Diagnocam the highest specificity in primary teeth. In contrast, the Trios 4 showed poorer results in all measured areas. This was also seen in permanent teeth, where the Diagnocam and Emerald S were superior to the Trios 4.

AUC values for IOS caries detection methods for threshold 1 ranged between 0.8 and >0.94, presenting excellent classification according to Hosmer et al. [19]. Comparable findings could be shown for threshold 2, with AUC values between >0.7 and >0.8/0.9.

**Figure 4:**
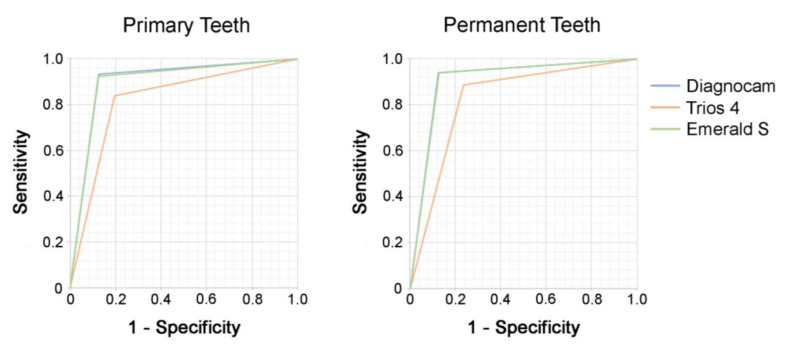
Receiver-operating-characteristic (ROC) curve analysis for primary teeth (**left**) and permanent teeth (**right**) for the investigated caries detection methods with threshold I (pooled data of sound (0) versus overall caries (pooled data of enamel caries (1) and dentin caries (2))).

**Figure 5:**
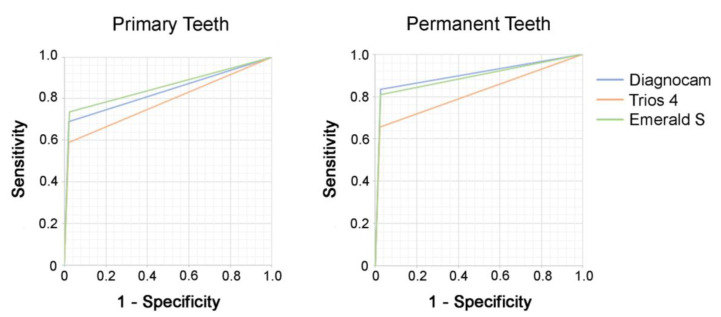
Receiver-operating-characteristic (ROC) curve analysis for primary teeth (**left**) and permanent teeth (**right**) for the investigated caries detection methods with threshold II (pooled data of sound (0) and enamel caries (1) versus dentin caries (2)).

Overall, except for specificity, the Trios 4 again showed the lowest values for all other parameters (Table 1, Figure 4 and Figure 5).

## 4. Discussion

The null hypothesis that there is no significant difference between visual examination and Diagnocam, Trios 4 or Emerald S in terms of reliability, sensitivity and specificity for the detection of enamel and dentinal caries lesions had to be rejected. Nevertheless, the Diagnocam showed in this clinical study the highest agreement with the reference standard visual examination in both primary and permanent teeth. Moreover, both IOSs also showed high agreement, with the Emerald S performing better than the Trios 4 in both dentitions.

Recent studies have described a wide range of methods for detecting occlusal caries [9,10]. Visual or visual–tactile inspection are common conventional methods for caries detection and classification in daily clinical practice and are still considered the gold standard for occlusal caries diagnosis. In addition, due to the described underestimation, the combination with the Diagnocam is suggested [8,13]. Regarding IOSs, there is no generally recognized IOS that is suitable for the detection of both occlusal and proximal caries to date [32]. A previous in vitro study showed that the IOS Emerald S is superior to the established gold standard of visual examination regarding the detection of occlusal caries lesions in permanent teeth. However, different results were found for primary teeth, where the IOS Trios 4 showed superior results compared to the Emerald S [5]. For this reason, the present study included primary molars in addition to permanent premolars and molars.

However, the use of IOSs also have disadvantages, such as the high error rate in the detection of enamel caries [33]. In the current study, the AUC of both evaluated methods was higher for more extensive caries lesions than initial lesions, which means that the IOS cannot be used as a tool to help decide whether to monitor and/or to proceed invasively. This aspect is also shown by other studies, which demonstrate that no absolute limit values can be defined for optical caries detection with fluorescence. Thus, the defined thresholds should only be seen as an indication of the relative caries lesion [32,34].

Regarding the limitations of this study, it should be noted that it was only possible to compare each method (Diagnocam, Trios 4 and Emerald S) with visual examination, i.e., it was only possible to show whether each occlusal caries detection method was better, worse or comparable to the reference method. Moreover, factors known to influence the accuracy of scanners in other applications, such as the choice of technology, ambient lighting, operator experience and the presence of existing restorations, were not considered [35]. Moreover, besides these aspects, assessment is very subjective, since it is primarily based on the experience of the clinician [34]. This diagnostic variability between clinicians leads to different decisions for the management of carious lesions, and several of them might be in the wrong direction [32,33].

Visual examination was carried out using the DMF-T/dmf-t index because this is the standard procedure in daily pediatric patient care. Therefore, the severity and caries activity could not be determined, which must be also considered a limitation of the present study. In new studies, the ICDAS would be preferred because it can track changes and progression of carious lesions [36].

Another limitation of the technologies used is the inability to determine the volume and depth of the carious lesion.

However, the potential of the Emerald S and Trios 4 in occlusal caries detection was proven in this clinical study, showing similar results as the Diagnocam and almost perfect agreement with the reference standard visual examination. This study points out the better performance of caries detection through near-infrared transillumination used in the Diagnocam and Emerald S than the fluorescence technology used in the Trios 4. This might be explained because porphyrins may be a useful marker for bacteria associated with dental caries but are not always linked to bacteria associated with dental caries [27]. In addition, other elements may also fluoresce at a wavelength of 415 nm. Therefore, further studies should analyze the influence of blood or saliva.

Although the Emerald S and Diagnocam are based on the same operating principle, near-infrared transillumination, the Emerald S uses the lowest wavelength (727 nm), followed by Diagnocam (780 nm), which seems to be advantageous for caries detection.

Therefore, IOSs, and in particular the Emerald S, appear to be a good alternative to the Diagnocam for the additional detection of occlusal lesions if they have no restorations. For clinical situations, the purchase of a Diagnocam could therefore be avoided if an IOS is already available.

It has to be discussed that both near-infrared transillumination and fluorescence technology display false-positive results of fillings or restorations. Therefore, the application of occlusal caries detection with IOSs is limited to natural teeth without any sealants or restorations. The present study only investigated occlusal caries lesions. However, clinical studies on proximal carious lesions in primary and permanent dentitions should be conducted as well.

The findings of this study are more likely to support the use of intraoral scanning to screen patients for caries, as part of serial examinations in kindergartens/schools or for visualization and/or teledentistry as previously suggested by Kopycka-Kedzierawski et al. [37]. Here, the role of the IOS could be an additional tool, which could certainly be helpful for this application. Today, IOSs do not allow the measurement of the depth or volume of carious lesions, which is already possible with the Canary system [38]. Therefore, further development of the caries detection tool in IOS is desirable as a perspective, perhaps using technologies other than near-infrared transillumination or fluorescence.

## 5. Conclusions

Within the limitations of this clinical study, it can be concluded that the IOS, at its current stage of development, has not yet reached the level of the gold standard, visual examination. However, in terms of technology, near-infrared transillumination seems to be superior to fluorescence.

At present, the IOS should be used as an additional detection tool and cannot be recommended as a basic tool for invasive/noninvasive therapy decisions in occlusal caries detection.

## Figures and Tables

**Table 1:** Caries detection methods regarding sensitivity, specificity and area-under-curve (AUC) values with receiver-operating-characteristic (ROC) curve analysis for threshold I (sound (0) versus overall caries (pooled data of enamel (1) and dentin caries (2))) and threshold II (pooled data of sound (0) and enamel caries (1) versus dentin caries (2)).

		Primary Teeth			Permanent Teeth		
Threshold	Caries Detection Method	Sensitivity	Specificity	AUC	Sensitivity	Specificity	AUC
I	Diagnocam	0.934	0.873	0.903	0.938	0.876	0.907
	Trios 4	0.840	0.804	0.822	0.887	0.765	0.826
	Emerald S	0.925	0.876	0.900	0.938	0.872	0.907
II	Diagnocam	0.691	0.979	0.835	0.836	0.974	0.905
	Trios 4	0.589	0.980	0.784	0.655	0.977	0.816
	Emerald S	0.737	0.976	0.857	0.810	0.976	0.893

## Data Availability

The data sets in this article are available from the corresponding author upon reasonable request.
